# Assessing the overlap between immunisation and other essential health interventions in 92 low- and middle-income countries using household surveys: opportunities for expanding immunisation and primary health care

**DOI:** 10.1016/j.eclinm.2021.101196

**Published:** 2021-11-06

**Authors:** Thiago M Santos, Bianca O Cata-Preta, Tewodaj Mengistu, Cesar G Victora, Daniel R Hogan, Aluisio J D Barros

**Affiliations:** 1 International Center for Equity in Health, Federal University of Pelotas, Pelotas, Brazil; 2 Postgraduate Program in Epidemiology, Federal University of Pelotas, Pelotas, Brazil; 3 Gavi, the Vaccine Alliance, Geneva, Switzerland

**Keywords:** vaccination, Immunization, Child health, child care, prenatal care, developing countries, hand disinfection

## Abstract

**Background:**

Unvaccinated children may live in households with limited access to other primary health care (PHC) services, and routine vaccination services may provide the opportunity to bring caregivers into contact with the health system. We aimed to investigate the overlap between not being vaccinated and failing to receive other PHC services in low- and middle-income countries (LMICs).

**Methods:**

Using Demographic and Health Surveys (DHS) and Multiple Indicator Cluster Surveys (MICS) data between 2010-2019 from 92 LMICs, we analysed six vaccination indicators based on the bacille Calmette-Guérin (BCG), polio, diphtheria-pertussis-tetanus (DPT) and measles vaccines and their overlap with four other PHC indicators - at least four antenatal care (ANC) visits, institutional delivery, careseeking for common childhood illnesses or symptoms and place for handwashing in the home - in 211,141 children aged 12-23 months. Analyses were stratified according to wealth quintiles and World Bank income levels.

**Findings:**

Unvaccinated children and their mothers were systematically less likely to receive the other PHC interventions. These associations were particularly marked for 4+ ANC visits and institutional delivery and modest for careseeking behaviour. Our stratified analyses confirm a systematic disadvantage of unvaccinated children and their families with respect to obtaining other health services in all levels of household wealth and country income.

**Interpretation:**

We suggested that lack of vaccination goes hand in hand with missing out on other health interventions. This represents an opportunity for integrated delivery strategies that may more efficiently reduce inequalities in health service coverage.

**Funding:**

Bill & Melinda Gates Foundation, Gavi, the Vaccine Alliance, The Wellcome Trust, Associação Brasileira de Saúde Coletiva and Coordenação de Aperfeiçoamento de Pessoal de Nível Superior.

**Research in context**

**Evidence before this study**

The prevalence of unvaccinated children is still unacceptably high in many low- and middle-income countries (LMICs). There is a growing body of evidence that children from families without access to primary health care (PHC) services – such as institutional delivery, antenatal care and maternal vaccination – also tend to be less likely to be vaccinated. We searched PubMed and Web of Science and there were no studies with multiple countries that analysed the overlap between non-vaccination and PHC service coverage.

**Added value of this study**

We found that unvaccinated children and their families are less likely to receive antenatal care, deliver in a health facility, seek care for child illness, and have access to handwashing facilities in the home. We also present the degree to which those failures overlap and find these relationships to be consistent across country income groups and, within countries, according to household wealth quintiles.

**Implications of all the available evidence**

Families and communities that are being left out of vaccination and other PHC services represent compelling targets for integrated delivery strategies that may more efficiently reduce inequalities in health service coverage. Identifying zero-dose children could be an important first step towards identifying communities missing out on other PHC services.

Alt-text: Unlabelled box

## 1 Introduction

While coverage of new vaccines for children living in low- and middle-income countries (LMICs) increased during the 2010 decade, the coverage of basic vaccines has stagnated [1,2]. In 2019, the coverage of three doses of the DPT vaccine in children aged 12-23 months was 95% in high income countries, 87% in middle income and as low as 74% in low income countries [2]. A recent analysis showed that 7·7% of the pooled population of children aged 12-23 months in 92 LMICs (the same set of countries studied in this work) had not received a single dose of any of the four basic vaccines – DPT, BCG, polio (poliomyelitis), or MCV (measles containing vaccine) [3]. These children who have not received any routine vaccinations are referred to as “zero-dose children”. For operational purposes, the World Health Organization's Immunization Agenda 2030 (IA2030) defines zero-dose children as those children who have not received any doses of DPT-containing vaccine, and has adopted a target of reducing the number of zero-dose children by 50% by 2030 as compared to a 2019 baseline of 13·6 million. Between 2019 and 2020, the number of zero-dose children increased by almost 3·5 million globally due to the COVID-19 pandemic, highlighting even greater efforts will be required to restore and sustainably expanding routine immunisation services in the coming decade [4,5].

Despite the challenges posed to vaccine distribution and delivery, vaccination coverage remains higher than several of other essential maternal and child health interventions, such as antenatal and delivery care, oral rehydration for diarrhoea or antibiotics for pneumonia [4,6]. Concerns have been raised about the possibility that vertical, well-funded immunisation programs may detract from activities and funding that should be directed to broader primary health care programs targeting women and children. On the other hand, the advantages of vertical programs include greater service specialization, increased profile for high-priority diseases or services, better monitoring and accountability, and more rapid results in weak health systems [7,8].

The children being missed by vaccination services may live in households or communities with limited access to other PHC services, and vaccination may provide the opportunity to bring families into contact with the health system and receive other PHC services [9]. The reverse may also be true and contact with PHC services may provide increased opportunities for vaccination [10]. Therefore, the current focus on zero-dose children by international agencies and national governments raises an opportunity to expand vaccination coverage while creating synergies to improve access to other health services. For this purpose, it is essential to document the extent to which unvaccinated children and their families are also missing out on other key health interventions. Studies of how access to services and interventions are clustered at individual levels have been described as analyses of cocoverage [11]. Yet, the literature on this issue is scant. A multi-country analysis revealed that there was substantial overlap between full vaccination coverage for children and coverage of the four other interventions delivered to pregnant women and children (antenatal care, skilled birth attendance, postnatal care for the child and vitamin A supplementation) [10].  Nevertheless, in eight out of the 14 countries included in the analysis, the authors found that 50% or more of children who were in contact with health services for other interventions failed to be fully vaccinated.

In the present article, we analysed survey data from 92 LMICs to estimate how often children with no vaccinations and their families benefit from antenatal care, institutional delivery, careseeking behaviour for child illness, and having access to water and a cleansing agent for handwashing. We used several indicators for unvaccinated children, including children with no doses DPT, no doses of BCG, no doses of polio, and no doses of MCV, as well as no doses of any of the four vaccines. Given the current priority in the design of programs and policies, the results focused on unvaccinated children as defined by lack of DPT-containing vaccine as the main outcome, while also presenting results for children who failed to receive the other three basic vaccines and for fully immunised children.

## 2 Methods

### 2.1 Data sources and study sample

We analysed the most recent Demographic and Health Surveys (DHS) and Multiple Indicator Cluster Surveys (MICS) carried out between 2010 and 2019 in LMICs. DHS and MICS are nationally representative cross-sectional health surveys that employ comparable multi-stage sampling methods, indicator definitions and questionnaires [12].

Our study included all children aged 12-23 months in the sampled households. The exceptions were Moldova (2012) where we studied children aged 15-26 months, and Jamaica (2011), Ukraine (2012), Costa Rica (2011), Tunisia (2011), Bosnia and Herzegovina (2011), North Macedonia (2011), and Egypt (2014) where we studied children aged 18-29 months. The reason for the exceptions is that in these countries the measles vaccine is offered after the age 12 months, while most countries offer it at age 9-12 months. Therefore, for comparison purposes, we followed the published national report estimates of these countries. This resulted in 2,903 children aged 24-29 months in our sample, representing only 1·8% of the weighted total of children.

### 2.2 Vaccination data

Data on vaccines received were obtained from two sources: vaccination cards or, if the child did not have one or it was not available at the time of interview, the mother's or caregiver's report. The vaccines included in the analyses were DPT, BCG, polio and MCV, regarded as the basic vaccines in immunisation programs. Monovalent or combination vaccines were taken into account when estimating coverage of polio, DPT and measles vaccines.

Six vaccine indicators were calculated. The first four referred to the proportions of children who failed to receive any doses of an individual vaccine: no-DPT, no-BCG, no-polio and no-MCV children. The fifth indicator was the proportion of children who received zero doses for all four vaccines: children with no vaccinations. The sixth indicator was the proportion of children who were fully vaccinated, defined as those children who received at least one dose of BCG, one dose of MCV, three doses of polio and three doses of DPT. In accordance with the World Health Organization recommendations, we treated children with missing information on vaccines as not vaccinated. Polio doses given soon after birth were not considered [13].

### 2.3 Primary care and hygiene indicators

We selected three maternal and childcare coverage indicators, closely related to PHC: at least four antenatal care visits (4+ ANC visits), institutional delivery, and careseeking for common childhood illnesses or symptoms (careseeking behaviour). We also used one hygiene indicator: place for handwashing in the home (handwashing facility). While the definition of what constitutes PHC varies across sources, we refer to them as PHC indicators, for conciseness [14,15]. The selection was based on the importance of these indicators along the reproductive, maternal, neonatal and child health (RMNCH) continuum of care, from conception to childhood, including adequate access to hygiene. We also gave preference to indicators available in a large number of countries.

Four or more antenatal care visits was defined as the proportion of children whose mothers received at least four antenatal care visits (irrespective of provider training) during pregnancy. Institutional delivery involved delivery in any type of health facility. Careseeking behaviour was defined as the proportion of children with diarrhoea, suspected pneumonia (cough plus rapid or difficulty breathing) or fever in the two weeks prior to the interview for whom treatment was sought from an appropriate health provider. Categories of appropriate providers were defined by each study country, usually including primary health units, clinics, hospitals, but not a pharmacy, for example. Handwashing facility in the home was defined as the proportion of study children living in a household with a specific place with water and soap or other cleansing agent for handwashing. These are Sustainable Development Goals indicators [16]. Further information on the definitions and calculation is available at the International Center for Equity in Health website (www.equidade.org/indicators).

### 2.4 Statistical analysis

The analyses were carried out with Stata (StataCorp. 2019. Stata Statistical Software: Release 16. College Station, TX: StataCorp LLC) and R (R Core Team, 2020, version 4.0.2. R Foundation for Statistical Computing, Vienna, Austria). DHS and MICS samples are drawn in two stages, clusters (usually census tracts) and households. The design also includes strata which typically have different sampling fractions. All surveys include variables for weights, primary sampling units and strata. The analyses accounted for this complex survey design, using the *svy* commands in Stata and the survey package in R. Pooled results were weighted by the national populations of children aged 12 to 23 months in 2015 (the median year of the surveys included) irrespective of specific vaccine indicator age ranges. Population data was obtained from the World Bank Population Estimates and Projections [17].

We calculated the coverage of each of the four health indicators stratified by immunisation status. We also calculated the ratio of coverage of the four indicators between non-vaccinated and vaccinated children and a Fisher's exact test to assess the statistical significance of the difference in coverage. Between-country differences were investigated by stratifying results according to World Bank country income levels (low, lower-middle, and upper-middle income) [18].

To analyse within-country socioeconomic inequalities we used the wealth index provided by DHS and MICS with each database. The index is derived using principal component analysis based on household assets, characteristics of the dwelling and utilities (e.g. electricity) [19]. The final score is adjusted in a way to make it comparable between urban and rural areas, and for both together [20,21]. The households are then ranked from poorest to wealthiest and grouped into five groups of equal size, taking into account the number of residents. The first quintile represents the households with the poorest 20% of the sample, and the fifth quintile, the wealthiest 20%.

### 2.5 Ethical aspects

Ethical approval for the conduct of the surveys was obtained by the national institutions involved in data collection. All data used were anonymized. Since we used only secondary data from these surveys, this study did not require ethical approval.

### 2.6 Role of the funding source

Beyond the individual technical contributions of TM and DRH, Gavi employees, the funders of the study had no role in the study design, data analysis, data interpretation, or writing of the report. The corresponding author had full access to all the data in the study and had final responsibility for the decision to submit for publication.

## 3 Results

In total, 92 countries had available data on the four vaccines included in the present study. These countries encompassed 90% of all low-income, 73% of lower-middle and 46% of the upper middle-income countries in the world. The surveys analysed included data from 211,141 children, of whom 51·2% were male, 64·3% lived in rural areas and 66·5% were from lower-middle income countries (Table 1). Table 1 and Supplementary Tables S1 and S2 provide a description of the sample and data sources for all countries included in the analyses.

**Table 1:** Characteristics of the sample and immunisation and PHC indicators coverage, for 92 countries. Source: DHS and MICS, 2010-2019.

		Unweighted mean	Unweighted standard deviation	Weighted mean	Weighted standard deviation	Range		Median	Interquartile range	
						Lowest	Highest		P25	P75
Sex of the child	Female	48·8%	2·0%	48·8%	1·8%	43·4%	54·6%	48·9%	47·5%	49·9%
	Male	51·2%	2·0%	51·2%	1·8%	45·4%	56·6%	51·1%	50·1%	52·5%
Place of residence	Rural	58·0%	18·9%	64·3%	14·7%	11·8%	91·8%	61·7%	42·3%	71·7%
	Urban	42·0%	18·9%	35·7%	14·7%	8·2%	88·2%	38·3%	28·3%	57·7%
Immunisation indicators	No DPT	11·5%	12·3%	13·9%	10·8%	0·0%	72·7%	7·3%	3·4%	14·1%
	No BCG	9·6%	11·4%	12·1%	10·6%	0·2%	66·1%	5·1%	2·4%	11·9%
	No POLIO	11·3%	11·3%	11·9%	9·0%	0·0%	64·4%	7·7%	3·7%	16·3%
	No MCV	22·4%	14·0%	24·3%	12·6%	3·3%	74·1%	19·3%	12·2%	30·8%
	No vaccinations	6·4%	8·8%	7·7%	6·9%	0·0%	57·6%	2·9%	1·5%	8·4%
	FIC	63·2%	20·5%	59·9%	17·4%	6·0%	93·9%	67·1%	51·4%	79·2%
PHC indicators	4+ ANC visits	69·7%	21·0%	59·1%	18·7%	17·7%	98·4%	75·0%	53·3%	87·6%
	Institutional delivery	78·1%	21·9%	72·1%	20·0%	11·8%	100·0%	85·3%	65·1%	96·7%
	Careseeking behaviour	54·7%	13·1%	60·5%	14·5%	26·1%	87·0%	53·8%	45·2%	64·7%
	Handwashing facility	50·5%	32·2%	54·0%	26·9%	2·9%	99·6%	46·9%	20·1%	83·5%

The overall weighted proportion of no-DPT children was 13·9%, ranging from 0·0% to 72·7% across the studied countries. The weighted proportion of children with no vaccinations was 7·7%, ranging from 0·0% to 57·6%. MCV had the highest proportion of unvaccinated children, with 24·3% overall, ranging from 3·3% to 74·1%. The pooled, weighted, PHC coverage levels were 59·1% for 4+ ANC visits, 72·1% for institutional delivery, 60·5% for careseeking behaviour and 54·0% for handwashing facility (Table 1).

When coverage levels of the four PHC interventions were stratified according to the six immunisation indicators, we found that coverage of all interventions were associated with vaccination status (Table 2). We calculated the ratio of coverage between non-vaccinated and vaccinated (at least one dose) children. Mothers of no-DPT children are 46% less likely to have 4+ ANC visits compared to mothers of vaccinated children (p value < 0·001). Lack of any DTP vaccination was also associated with lower coverage of the other three interventions, with coverage of institutional delivery, careseeking behaviour and handwashing facilities 43%, 18% and 36% lower among no-DPT children (p value < 0·001 for all three indicators). For children with no vaccinations, coverages were 51%, 47%, 24%, and 36% lower for 4+ ANC visits, institutional delivery, careseeking behaviour and handwashing facility, respectively, when compared to vaccinated children (Table 2, p value < 0·001 for all four indicators).

**Table 2:** Coverage (%) of 4+ ANC visits, institutional delivery, careseeking behaviour and handwashing facility according to vaccination indicators and the respective coverage ratio. Source: DHS and MICS, 2010-2019.

Note: all comparisons between unvaccinated and vaccinated children presented a p value < 0.001 (Fisher's exact test).

The strongest associations (highlighted in red in Table 2) were found for no vaccinations and no BCG in relation to 4+ ANC visits, with a 51% lower coverage (p value < 0·001). Similarly, no-BCG children and their mothers were 51% less likely to access institutional delivery, 21% less likely to seek care and 42% less likely to have access to a handwashing facility in the household, compared to children vaccinated with BCG and their mothers (p value < 0·001 for all three indicators). No-polio and no-MCV children had weaker associations for all PHC interventions, but fairly similar results among themselves (Table 2).

The results for full immunisation coverage, a desirable situation, showed that fully immunized children and their mothers were more likely to receive any of the four PHC interventions compared to children who were not fully immunized, from 15% higher careseeking behaviour to 39% higher coverage of 4+ ANC visits (Table 2, p value < 0·001 for the two indicators).

**Figure 1:**
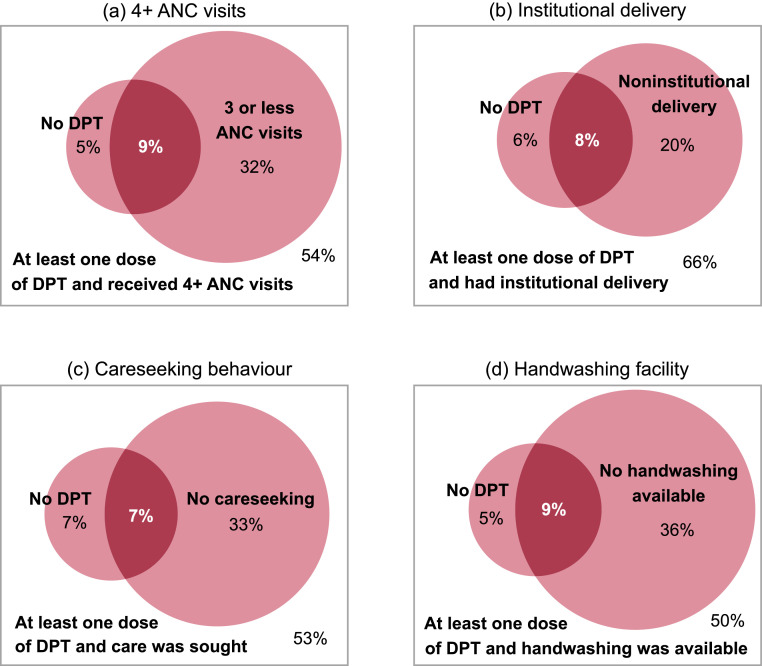
Intersection between no DPT and lack of 4+ ANC visits, institutional delivery, careseeking behaviour and handwashing facility. Weighted average values from 92 national surveys. DPT – diphtheria, pertussis, tetanus vaccine. ANC – antenatal care. Darker pink refers to the intersection between no DPT and lack of PHC services. Panel (a): the bigger circumference represents the percentage of children whose mothers received three or less antenatal care visits during pregnancy. The smaller circumference indicates the percentage of children who did not receive any doses of DPT vaccine. The intersection, in darker pink, represents the percentage of children who did not receive any doses of DPT vaccine and whose mothers received three or less antenatal care visits. The rectangle depicts the percentage of children who received at least one dose of DPT and whose mother received at least four antenatal care visits. Panel (b): the bigger circumference represents the percentage of children who had noninstitutional delivery. The smaller circumference indicates the percentage of children who did not receive any doses of DPT vaccine. The intersection, in darker pink, represents the percentage of children who did not receive any doses of DPT vaccine and had noninstitutional delivery. The rectangle depicts the percentage of children who received at least one dose of DPT and had institutional delivery. Panel (c): the bigger circumference represents the percentage of children with diarrhoea, suspected pneumonia or fever for whom no treatment was sought from an appropriate health provide. The smaller circumference indicates the percentage of children who did not receive any doses of DPT vaccine. The intersection, in darker pink, represents the percentage of children who did not receive any doses of DPT vaccine and for whom no treatment was sought. The rectangle depicts the percentage of children who received at least one dose of DPT and for whom treatment was sought. Panel (d): the bigger circumference represents the percentage of children living in a household with no handwashing facility. The smaller circumference indicates the percentage of children who did not receive any doses of DPT vaccine. The intersection, in darker pink, represents the percentage of children who did not receive any doses of DPT vaccine and live in a household with no handwashing facility. The rectangle depicts the percentage of children who received at least one dose of DPT and live in a household with handwashing facility.

We also looked at the overlap between no DPT and PHC interventions (Figure 1 and Table S3). Overall, 9·1% (95% CI 8·8%;9·4%) of children were no DPT and their mothers did not receive 4+ ANC visits. This proportion was similar for non-institutional delivery (7·8%, 95% CI 7·5%;8·1%), no careseeking behaviour (7·0%, 95% CI 6·6%;7·4%) and no handwashing facility (8·6%, 95% CI 8·2%;8·9%). The proportion of children who had received the interventions and at least one dose of DPT varied between 50·4% (95% CI 49·9%;51·0%) and 66·0% (95% CI 65·5%;66·5%) for each of the interventions (Figure 1 and Table S3). The overlap between no vaccinations and PHC interventions is presented in Supplementary Figure S1.

The above relationships were qualitatively similar when looking at lack of vaccination for other antigens. The overlap between no BCG, no polio and no MCV with not receiving PHC interventions is presented in Table S3. Overall, between 6·4% (95% CI 6·1%;6·8%) and 8·2% (95% CI 8·0%;8·5%) of children did not receive BCG nor a specific PHC intervention. For polio, they were between 5·6% (95% CI 5·3%;5·9%) and 7·3% (95% CI 7·0%;7·5%), and for MCV, between 11·2% (95% CI 10·9%;11·5%) and 14·0% (95% CI 13·6%;14·3%).

**Figure 2:**
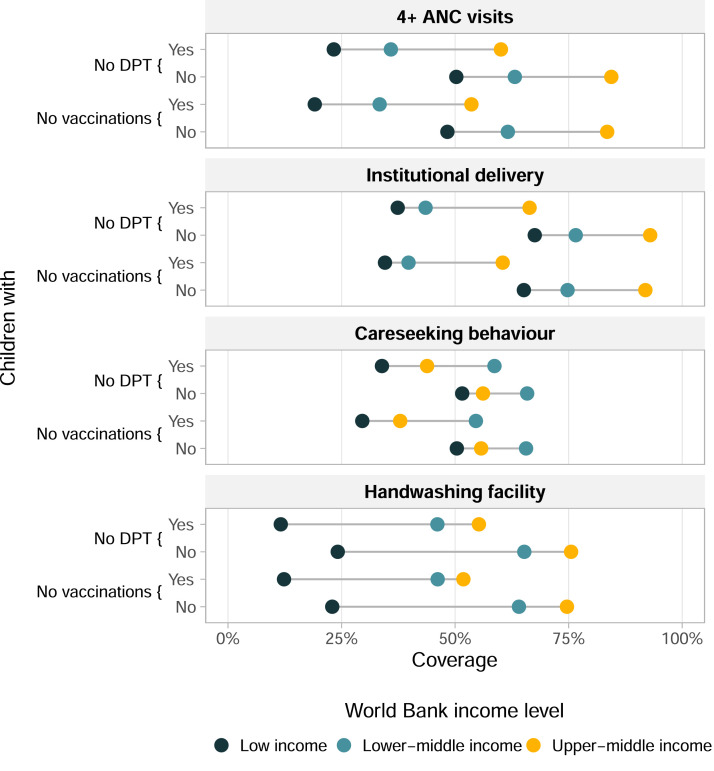
Coverage of PHC interventions according to vaccination status of the child, stratified by World Bank country income groups, averaged over 92 countries. Each dot corresponds to a country income group. PHC – primary health care. DHS – Demographic and Health Surveys. MICS – Multiple Indicator Cluster and Surveys. DPT – diphtheria, pertussis, tetanus vaccine. No vaccination refers to lack of any BCG, DPT, polio and MCV doses. ANC – antenatal care. The x-axis shows the vaccination coverage. In the y-axis are shown children who received no DPT vaccination and who received at least one dose of DPT vaccine, also children who received no vaccinations and who received at least one dose of BCG, DPT, polio, or MCV.

Figure 2 shows the weighted average coverage of each PHC interventions for no-DPT children and those with at least one dose of DPT as well as for children with no vaccinations and those with at least one dose of any vaccine, with countries grouped by World Bank income level (Supplementary Table S4 shows the sample sizes in each group). Regardless of immunisation status, coverage of the PHC interventions increased with country income level, with differences as high as 50 percentage points, except for careseeking behaviour for which upper-middle income countries had an intermediate level of coverage and lower-middle income countries the highest coverage (Figure 2). No-DPT children had lower coverage of the PHC interventions in all cases, compared to children with at least one dose of DPT. The differences were particularly marked for 4+ ANC visits and institutional delivery. For institutional delivery, mothers of children with at least one dose of DPT in low-income countries had comparable coverage to mothers of no-DPT children in upper-middle income countries (70%, approximately). Careseeking behaviour was the indicator with the least marked differences among the three country income groups and between no-DPT children and those with at least one dose of DPT (Figure 2).

**Figure 3:**
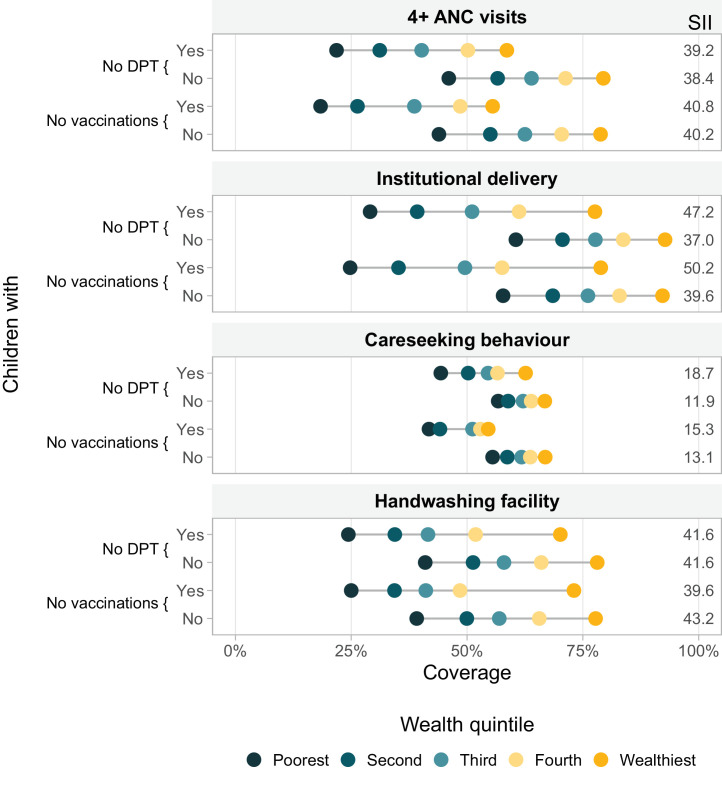
Coverage of PHC interventions according to vaccination status of the child, stratified by country-specific wealth quintiles, averaged over 91 countries. Each dot represents a wealth quintile. PHC – primary health care. DHS – Demographic and Health Surveys. MICS – Multiple Indicator Cluster and Surveys. DPT – diphtheria, pertussis, tetanus vaccine. No vaccination refers to lack of any BCG, DPT, polio and MCV doses. ANC – antenatal care. SII – Slope Index of Inequality. The x-axis shows the vaccination coverage. In the y-axis (left side) are shown children who received no DPT vaccination and who received at least one dose of DPT vaccine, also children who received no vaccinations and who received at least one dose of BCG, DPT, polio, or MCV. On the right side of the figure is shown the SII - Note: Cuba was excluded due to lack of wealth index information. The Slope Index of Inequality (SII) is a measurement of absolute inequality that represents the predicted difference in service coverage between the extremes of the wealth distribution. The farther values are from zero, the larger the absolute inequalities.

In Figure 3, we present the weighted average coverage of the four PHC indicators for no-DPT children and those with at least one dose of DPT, as well as for children with no vaccinations and those with at least one dose of any of the four vaccines, by wealth quintiles. Again, in every case, coverage with other services was lower for no-DPT children, compared to those with at least one dose of DPT belonging to the same wealth quintile. Also, coverage was always higher for children from wealthier than for those from poorer families. For institutional delivery and careseeking behaviour, inequalities among no-DPT children were markedly higher than among children with at least one dose of DPT, as evidenced by the slope indices of inequality (SIIs) being some 7 to 10 percentage points larger. While the mothers of the poorest no-DPT children had an institutional delivery coverage of 29·1% (95% CI 27·4%;30·8%), the mothers of the poorest children with at least one dose of DPT had a 60.5% (95% CI 59·5%;61·6%) coverage, representing a staggering absolute inequality of 31·4 (95% CI 29·4;33·6) percentage points. This absolute inequality between the poorest children were 24·2 (95% CI 22·3;26·1), 16·6 (95% CI 14·6;18·6) and 12·4 (95% CI 8·6;16·2) percentage points for 4+ ANC visits, handwashing facility and careseeking behaviour, respectively (Figure 3).

The results in Figures 2 and 3 were almost identical for children with no vaccinations and no-DPT, despite the proportion of no-DPT children being nearly twice that of children with no vaccinations (Table 1).

## 4 Discussion

Our analysis covers 92 countries and includes more than 200 thousand children and mothers, making it the largest multicountry analysis of co-coverage between vaccination and health interventions to date. We compare the coverage of health interventions according to vaccination status, using six indicators for vaccine coverage. Unvaccinated children and their mothers were systematically less likely to receive the four health interventions studied – 4+ ANC visits, institutional delivery, careseeking behaviour and handwashing facility in the home, compared to children who had received at least one dose. The comparisons showed substantial differences, with no-DPT children being up to 46% less likely to receive PHC interventions (Table 2).

The slightly stronger associations between having no vaccinations and PHC interventions compared to no DPT is not surprising given the no vaccinations indicator is stricter, and the number of children with no vaccinations is much smaller, comprising a select group of children who accumulate a larger degree of disadvantage. On the other hand, it is important that the no-DPT indicator presents a very similar set of results given it is easier to estimate from routine data and its measurement does not depend on survey data. Also, no DPT helps to identify children with limited access to health services since it is offered almost exclusively through routine immunisation touchpoints required for ensuring full immunisation, including vaccination against other diseases like pneumococcal pneumonia and rotavirus.

Coverage of maternal health care services were the most highly correlated with immunisation among the interventions considered in the analysis. While this association was highest for BCG vaccination, other vaccines, despite not being delivered at the time of birth in a health facility, showed similar patterns, with DPT having the highest associations of the other three vaccines. Co-coverage between immunisation and careseeking for child illness were correlated, with children with no vaccinations being less likely to seek care for suspected illness than children who had received at least some vaccination, but to a lesser degree than maternal health care services and handwashing facilities in the home. The reason for the weaker association for careseeking is unclear. This pattern may point to the dynamics around demand for health services, wherein caregivers are more likely to obtain services when faced with the emergency of an acute illness, as compared to preventive services like vaccination.

Coverage of health interventions decreased with country income level and with household wealth within countries, consistent with previous findings [22], [23], [24]. Our analyses assessing these patterns conditional on vaccination status illustrate a systematic disadvantage for unvaccinated children, for all subgroups and all interventions. Generally, no-DPT children and their families are worse off than children with at least one dose of DPT by approximately the same gap in coverage for all subgroups of country income level and household wealth (Figures 2 and 3). The combination of poverty with no DPT resulted in exceedingly low coverage with the PHC interventions, a pattern less marked for careseeking behaviour.

This analysis identifies the most disadvantaged households that are missing out on immunisation and other services, while also highlighting other households that receive one service but not the other and therefore represent missed opportunities for sustained contact with the health system. Approaches to integrating immunisation with other PHC services need to be tailored to the local context. One example of successful integration between services is the application of the “Reach Every District” strategy in the Byanzurkh District in Mongolia. Based on the assessment of program planners that the disadvantaged households shared similar barriers for service access, a package of maternal, newborn, and child health services, as well as water and sanitation was implemented. It included demographic surveys, registration of difficult-to-reach populations and mobile services [25].

Some limitations of our work should be mentioned. These results are not representative of all LMICs and given the focus of survey initiatives on poorer countries with worse health indicators, low-income countries are more represented than middle- or upper-middle-income countries. Non-vaccination assignment is somewhat dependent on missing information for some countries, as children with unknown vaccination status were considered as non-vaccinated. This is unlikely to affect the main results, given that only 0·7% (95% CI 0·6%;0·8%) of all children in our sample had missing information for DPT and 1·3% (95% CI 1·2%;1·4%) for any vaccine. Additionally, vaccination status was established using the mother's report when the vaccination card was unavailable. The report is subject to recall bias and might overestimate no vaccination prevalence. We used the wealth score, a relative indicator based on assets, to classify households according to wealth. This approach classifies the poorest in a poor country in the same category as the poorest in a richer country, while absolute levels of poverty may be quite different. However, we are more interested in the situation of the poorest in a given context – in order to assess the equitable distribution of immunisation services within a country – than in how much access to vaccines a given level of wealth measured in dollars can buy [26]. Children with no vaccinations and their mothers are clearly worse off in regard to other health interventions, and vice versa. Our analysis does not consider the direction of the associations, as we are solely assessing co-coverage using vaccination status as the basis for comparisons. Finally, the standard errors and associated confidence intervals might be underestimated given that between country variability was not taken into account, what could be done using multilevel models. We chose a simpler analytical approach to make the results more accessible for a wider audience.

We showed that lack of vaccination goes hand in hand with missing out on other health interventions, and that this pattern holds across country income groups and within countries across household wealth quintiles. Zero-dose children and their families are less likely to receive other PHC services in all contexts. This represents an opportunity for joining the forces of immunisation programs with other primary health care services. Identifying where and who zero-dose children and their families are could help identify communities missing out on other health services, with integrated delivery approaches potentially offering more efficient and effective means of increasing coverage of immunisation and other essential health services.

## Declaration of Competing Interest

TM and DHR are employed by Gavi, the Vaccine Alliance, sponsor of this research. They had total freedom to express their views which do not necessarily reflect those of Gavi, the Vaccine Alliance. All the other authors, TMS, BCP, CGV and AJDB, declare that they have no known competing financial interests or personal relationships that could have influenced the work reported in this paper.

## Appendix Supplementary materials

Image, application 1

Image, application 2
