# Supplementary material for: Assessing the overlap between immunisation and other essential health interventions in 92 low- and middle-income countries using household surveys: opportunities for expanding immunisation and primary health care
Source: eClinicalMedicine. 2021 Nov 6;42:101196. doi: 10.1016/j.eclinm.2021.101196 (PMC8585628; doi:10.1016/j.eclinm.2021.101196)
Supplement: Supplementary file 1 [file mmc1.docx]

# Caption for supplementary material

Caption for: Supplementary materials.docx

Supplementary Table 1. Country, year, data source and World Bank income group in the median year (2015) of the surveys included in the sample

Supplementary Table 2. Sample characteristics. Source: DHS and MICS, 2010-2019

Supplementary Table 3 – Co-coverage estimates of vaccination indicators prevalence and intervention coverage. Source: DHS and MICS, 2010-2019

Supplementary Table 4. Sample sizes. Source: DHS and MICS, 2010-2019

Supplementary Figure 1 – Intersection between having no vaccinations and lack of 4+ ANC visits, institutional delivery, careseeking behaviour and handwashing facility. Weighted average values from 92 national surveys. Source: DHS and MICS, 2010-2019

Caption: No vaccinations refer to lack of any BCG, DPT, polio and MCV. ANC – antenatal care. Darker pink refers to the intersection between no DPT and lack of PHC services. Panel (a): the bigger circumference represents the percentage of children whose mothers received three or less antenatal care visits during pregnancy. The smaller circumference indicates the percentage of children who received no vaccinations. The intersection, in darker pink, represents the percentage of children who received no vaccinations and whose mothers received three or less antenatal care visits. The rectangle depicts the percentage of children who received at least one dose of BCG, DPT, polio, or MCV and whose mother received at least four antenatal care visits. Panel (b): the bigger circumference represents the percentage of children who had noninstitutional delivery. The smaller circumference indicates the percentage of children who received no vaccinations. The intersection, in darker pink, represents the percentage of children who received no vaccinations and had noninstitutional delivery. The rectangle depicts the percentage of children who received at least one dose of BCG, DPT, polio, or MCV and had institutional delivery. Panel (c): the bigger circumference represents the percentage of children with diarrhoea, suspected pneumonia or fever for whom no treatment was sought from an appropriate health provide. The smaller circumference indicates the percentage of children who received no vaccinations. The intersection, in darker pink, represents the percentage of children who received no vaccinations and for whom no treatment was sought. The rectangle depicts the percentage of children who received at least one dose of BCG, DPT, polio, or MCV and for whom treatment was sought. Panel (d): the bigger circumference represents the percentage of children living in a household with no handwashing facility. The smaller circumference indicates the percentage of children who received no vaccinations. The intersection, in darker pink, represents the percentage of children who received no vaccinations and live in a household with no handwashing facility. The rectangle depicts the percentage of children who received at least one dose of BCG, DPT, polio, or MCV and live in a household with handwashing facility.
