# Supplementary material for: Assessing the overlap between immunisation and other essential health interventions in 92 low- and middle-income countries using household surveys: opportunities for expanding immunisation and primary health care
Source: eClinicalMedicine. 2021 Nov 6;42:101196. doi: 10.1016/j.eclinm.2021.101196 (PMC8585628; doi:10.1016/j.eclinm.2021.101196)
Supplement: Supplementary file 2 [file mmc2.docx]

**Supplementary materials**

**Supplementary Table 1. Country, year, data source and World Bank income group in the median year (2015) of the surveys included in the sample**

| **Country** | **Year** | **Source** | **World Bank income group** |
| --- | --- | --- | --- |
| Afghanistan | 2015 | DHS | Low income |
| Algeria | 2012 | MICS | Upper-middle income |
| Angola | 2015 | DHS | Upper-middle income |
| Armenia | 2015 | DHS | Lower-middle income |
| Bangladesh | 2014 | DHS | Lower-middle income |
| Belize | 2015 | MICS | Upper-middle income |
| Benin | 2017 | DHS | Low income |
| Bosnia and Herzegovina | 2011 | MICS | Upper-middle income |
| Burkina Faso | 2010 | DHS | Low income |
| Burundi | 2016 | DHS | Low income |
| CAR | 2010 | MICS | Low income |
| Cambodia | 2014 | DHS | Lower-middle income |
| Cameroon | 2014 | MICS | Lower-middle income |
| Chad | 2014 | DHS | Low income |
| Colombia | 2010 | DHS | Upper-middle income |
| Comoros | 2012 | DHS | Low income |
| Congo Brazzaville | 2014 | MICS | Lower-middle income |
| Congo, Democratic Republic | 2017 | MICS | Low income |
| Costa Rica | 2011 | MICS | Upper-middle income |
| Côte d’Ivoire | 2016 | MICS | Lower-middle income |
| Cuba | 2014 | MICS | Upper-middle income |
| Dominican Republic | 2014 | MICS | Upper-middle income |
| Egypt | 2014 | DHS | Lower-middle income |
| El Salvador | 2014 | MICS | Lower-middle income |
| Eswatini | 2014 | MICS | Lower-middle income |
| Ethiopia | 2016 | DHS | Low income |
| Gabon | 2012 | DHS | Upper-middle income |
| Gambia | 2018 | MICS | Low income |
| Ghana | 2017 | MICS | Lower-middle income |
| Guatemala | 2014 | DHS | Lower-middle income |
| Guinea | 2018 | DHS | Low income |
| Guinea Bissau | 2014 | MICS | Low income |
| Guyana | 2014 | MICS | Upper-middle income |
| Haiti | 2016 | DHS | Low income |
| Honduras | 2011 | DHS | Lower-middle income |
| India | 2015 | DHS | Lower-middle income |
| Indonesia | 2017 | DHS | Lower-middle income |
| Iraq | 2018 | MICS | Upper-middle income |
| Jamaica | 2011 | MICS | Upper-middle income |
| Jordan | 2017 | DHS | Upper-middle income |
| Kazakhstan | 2015 | MICS | Upper-middle income |
| Kenya | 2014 | DHS | Lower-middle income |
| Kiribati | 2018 | MICS | Lower-middle income |
| Kosovo | 2013 | MICS | Lower-middle income |
| Kyrgyzstan | 2018 | MICS | Lower-middle income |
| Laos | 2017 | MICS | Lower-middle income |
| Lesotho | 2018 | MICS | Lower-middle income |
| Liberia | 2013 | DHS | Low income |
| Madagascar | 2018 | MICS | Low income |
| Malawi | 2015 | DHS | Low income |
| Maldives | 2016 | DHS | Upper-middle income |
| Mali | 2018 | DHS | Low income |
| Mauritania | 2015 | MICS | Lower-middle income |
| Mexico | 2015 | MICS | Upper-middle income |
| Moldova | 2012 | MICS | Lower-middle income |
| Mongolia | 2018 | MICS | Lower-middle income |
| Montenegro | 2013 | MICS | Upper-middle income |
| Mozambique | 2015 | DHS | Low income |
| Myanmar | 2015 | DHS | Lower-middle income |
| Namibia | 2013 | DHS | Upper-middle income |
| Nepal | 2016 | DHS | Low income |
| Niger | 2012 | DHS | Low income |
| Nigeria | 2018 | DHS | Lower-middle income |
| North Macedonia | 2011 | MICS | Upper-middle income |
| Pakistan | 2017 | DHS | Lower-middle income |
| Panama | 2013 | MICS | Upper-middle income |
| Papua New Guinea | 2016 | DHS | Lower-middle income |
| Paraguay | 2016 | MICS | Upper-middle income |
| Peru | 2018 | DHS | Upper-middle income |
| Philippines | 2017 | DHS | Lower-middle income |
| Rwanda | 2014 | DHS | Low income |
| São Tomé and Príncipe | 2014 | MICS | Lower-middle income |
| Senegal | 2017 | DHS | Low income |
| Serbia | 2014 | MICS | Upper-middle income |
| Sierra Leone | 2017 | MICS | Low income |
| South Africa | 2016 | DHS | Upper-middle income |
| South Sudan | 2010 | MICS | Low income |
| State of Palestine | 2014 | MICS | Lower-middle income |
| Sudan | 2014 | MICS | Lower-middle income |
| Tajikistan | 2017 | DHS | Lower-middle income |
| Tanzania | 2015 | DHS | Low income |
| Thailand | 2015 | MICS | Upper-middle income |
| Timor-Leste | 2016 | DHS | Lower-middle income |
| Togo | 2017 | MICS | Low income |
| Tunisia | 2018 | MICS | Lower-middle income |
| Turkmenistan | 2015 | MICS | Upper-middle income |
| Uganda | 2016 | DHS | Low income |
| Ukraine | 2012 | MICS | Lower-middle income |
| Vietnam | 2013 | MICS | Lower-middle income |
| Yemen | 2013 | DHS | Lower-middle income |
| Zambia | 2018 | DHS | Lower-middle income |
| Zimbabwe | 2019 | MICS | Low income |

**Supplementary Table 2. Sample characteristics. Source: DHS and MICS, 2010-2019**

| **Characteristic** | **Level** | **n** | **Prevalence** |
| --- | --- | --- | --- |
| Sex | Male | 108034 | 51·2% |
|  | Female | 103107 | 48·8% |
| Age group | 12-14 months | 54495 | 25·8% |
|  | 15-17 months | 52578 | 24·9% |
|  | 18-20 months | 52381 | 24·5% |
|  | 21-23 months | 48784 | 23·0% |
|  | 24-29 months | 2903 | 1·8% |
| Area of residence | Urban | 73974 | 35·7% |
|  | Rural | 137167 | 64·3% |
| Wealth quintiles | First (Poorest) | 54523 | 22·9% |
|  | Second | 47117 | 21·3% |
|  | Third | 42049 | 20·5% |
|  | Fourth | 36277 | 19·0% |
|  | Fifth (Richest) | 30014 | 16·4% |
| Income group | Low income | 60145 | 22·1% |
|  | Lower-middle income | 111924 | 66·5% |
|  | Upper-middle income | 39072 | 11·4% |

**Supplementary Table 3 – Co-coverage estimates of vaccination indicators prevalence and intervention coverage. Source: DHS and MICS, 2010-2019**

|  |  | **4+ ANC visits** | | **Institutional delivery** | | **Careseeking behaviour** | | **Handwashing facility** | |
| --- | --- | --- | --- | --- | --- | --- | --- | --- | --- |
| **Vaccine** | | **Received** | **Not received** | **Received** | **Not received** | **Received** | **Not received** | **Received** | **Not received** |
| No vaccinations | Yes | 2·3 | 5·2 | 3·0 | 4·6 | 3·2 | 3·8 | 2·8 | 4·8 |
|  | No | 56·7 | 35·8 | 69·0 | 23·4 | 56·6 | 36·4 | 52·7 | 39·7 |
| No BCG | Yes | 3·7 | 8·2 | 4·5 | 7·5 | 6·1 | 6·4 | 4·0 | 7·8 |
|  | No | 55·3 | 32·8 | 67·5 | 20·5 | 53·7 | 33·8 | 51·5 | 36·7 |
| No POLIO | Yes | 4·5 | 7·3 | 5·9 | 5·9 | 5·5 | 5·6 | 4·7 | 7·0 |
|  | No | 54·5 | 33·8 | 66·1 | 22·1 | 54·3 | 34·6 | 50·8 | 37·5 |
| No DPT | Yes | 4·7 | 9·1 | 6·0 | 7·8 | 7·1 | 7·0 | 5·1 | 8·6 |
|  | No | 54·3 | 31·9 | 66·0 | 20·2 | 52·7 | 33·2 | 50·4 | 35·9 |
| No MCV | Yes | 10·4 | 14·0 | 13·0 | 11·2 | 13·3 | 12·1 | 10·1 | 13·8 |
|  | No | 48·6 | 27·1 | 59·0 | 16·8 | 46·5 | 28·1 | 45·4 | 30·7 |
| Fully immunized | Yes | 39·8 | 20·1 | 47·8 | 12·2 | 37·7 | 22·1 | 37·3 | 23·1 |
|  | No | 19·2 | 20·9 | 24·2 | 15·8 | 22·1 | 18·1 | 18·2 | 21·4 |

**Supplementary Table 4. Sample sizes. Source: DHS and MICS, 2010-2019**

| **PHC indicators** | | **Vaccination indicators** | | **Low income** | **Lower-middle income** | **Upper-middle income** |
| --- | --- | --- | --- | --- | --- | --- |
| All children | - | No vaccinations | Yes | 6821 | 8488 | 1651 |
|  |  |  | No | 53324 | 103436 | 37421 |
|  |  | No DPT | Yes | 11900 | 14532 | 3482 |
|  |  |  | No | 48245 | 97392 | 35590 |
| 4+ ANC visits | Received | No vaccinations | Yes | 1080 | 2373 | 751 |
|  |  |  | No | 25345 | 58749 | 29043 |
|  |  | No DPT | Yes | 2354 | 4479 | 1841 |
|  |  |  | No | 24071 | 56643 | 27953 |
|  | Not received | No vaccinations | Yes | 5169 | 5420 | 695 |
|  |  |  | No | 25422 | 38313 | 5708 |
|  |  | No DPT | Yes | 8714 | 8952 | 1313 |
|  |  |  | No | 21877 | 34781 | 5090 |
| Institutional delivery | Received | No vaccinations | Yes | 1704 | 3335 | 893 |
|  |  |  | No | 35060 | 76636 | 32389 |
|  |  | No DPT | Yes | 3521 | 6458 | 2199 |
|  |  |  | No | 33243 | 73513 | 31083 |
|  | Not received | No vaccinations | Yes | 4679 | 4960 | 663 |
|  |  |  | No | 17399 | 25210 | 3016 |
|  |  | No DPT | Yes | 7805 | 7742 | 1118 |
|  |  |  | No | 14273 | 22428 | 2561 |
| Careseeking behaviour | Received | No vaccinations | Yes | 596 | 1279 | 191 |
|  |  |  | No | 12208 | 20641 | 6705 |
|  |  | No DPT | Yes | 1355 | 2534 | 494 |
|  |  |  | No | 11449 | 19386 | 6402 |
|  | Not received | No vaccinations | Yes | 1495 | 1370 | 247 |
|  |  |  | No | 10492 | 12747 | 4758 |
|  |  | No DPT | Yes | 2624 | 2344 | 544 |
|  |  |  | No | 9363 | 11773 | 4461 |
| Handwashing facility | Received | No vaccinations | Yes | 595 | 3458 | 568 |
|  |  |  | No | 8818 | 58237 | 16609 |
|  |  | No DPT | Yes | 1091 | 6094 | 1316 |
|  |  |  | No | 8322 | 55601 | 15861 |
|  | Not received | No vaccinations | Yes | 4391 | 4190 | 660 |
|  |  |  | No | 35837 | 34878 | 5686 |
|  |  | No DPT | Yes | 7772 | 6997 | 1181 |
|  |  |  | No | 32456 | 32071 | 5165 |

**Supplementary Figure 1 – Intersection between having no vaccinations and lack of 4+ ANC visits, institutional delivery, careseeking behaviour and handwashing facility. Weighted average values from 92 national surveys. Source: DHS and MICS, 2010-2019**

**
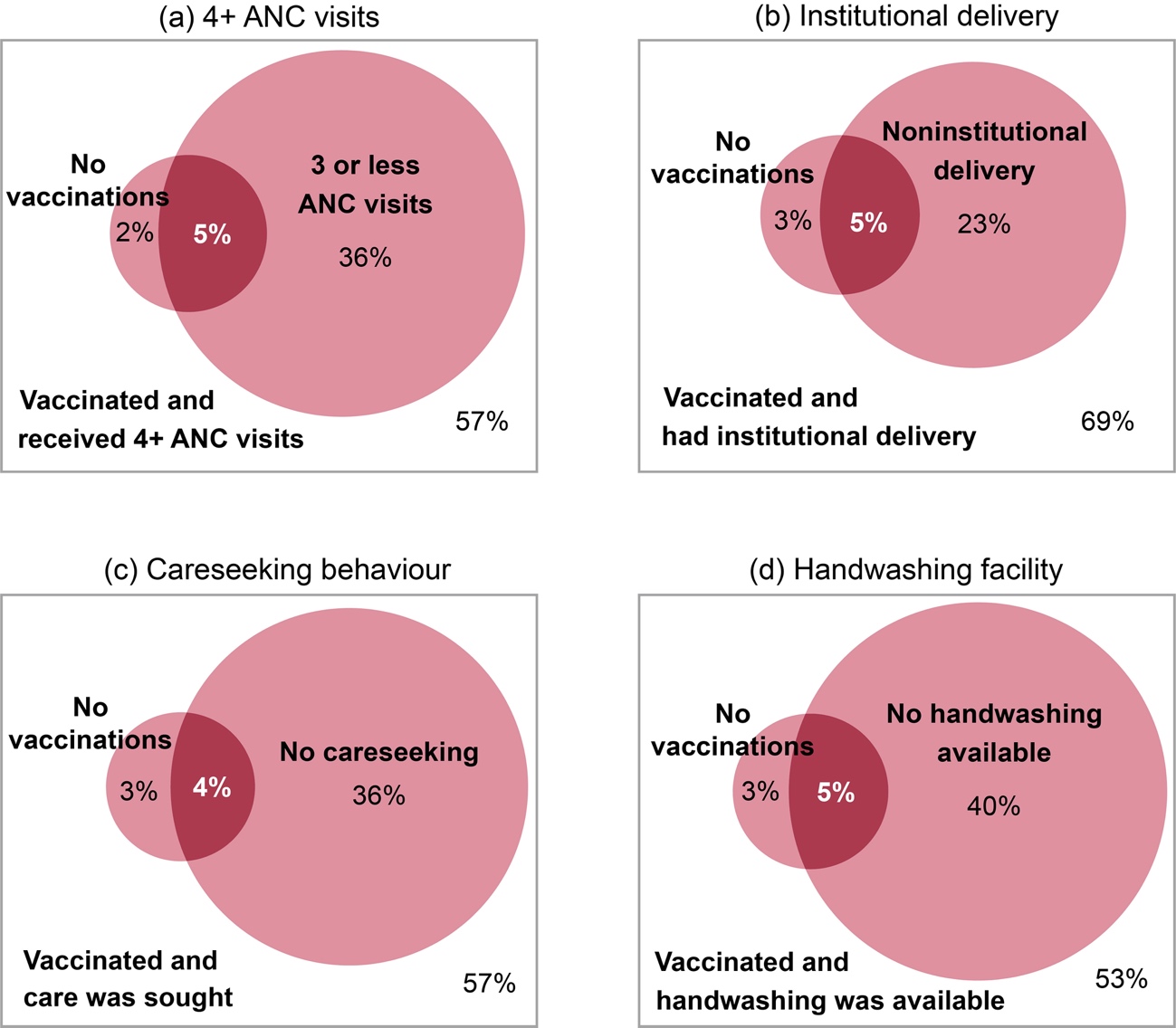
**

Caption: No vaccinations refer to lack of any BCG, DPT, polio and MCV. ANC – antenatal care. Darker pink refers to the intersection between no DPT and lack of PHC services. Panel (a): the bigger circumference represents the percentage of children whose mothers received three or less antenatal care visits during pregnancy. The smaller circumference indicates the percentage of children who received no vaccinations. The intersection, in darker pink, represents the percentage of children who received no vaccinations and whose mothers received three or less antenatal care visits. The rectangle depicts the percentage of children who received at least one dose of BCG, DPT, polio, or MCV and whose mother received at least four antenatal care visits. Panel (b): the bigger circumference represents the percentage of children who had noninstitutional delivery. The smaller circumference indicates the percentage of children who received no vaccinations. The intersection, in darker pink, represents the percentage of children who received no vaccinations and had noninstitutional delivery. The rectangle depicts the percentage of children who received at least one dose of BCG, DPT, polio, or MCV and had institutional delivery. Panel (c): the bigger circumference represents the percentage of children with diarrhoea, suspected pneumonia or fever for whom no treatment was sought from an appropriate health provide. The smaller circumference indicates the percentage of children who received no vaccinations. The intersection, in darker pink, represents the percentage of children who received no vaccinations and for whom no treatment was sought. The rectangle depicts the percentage of children who received at least one dose of BCG, DPT, polio, or MCV and for whom treatment was sought. Panel (d): the bigger circumference represents the percentage of children living in a household with no handwashing facility. The smaller circumference indicates the percentage of children who received no vaccinations. The intersection, in darker pink, represents the percentage of children who received no vaccinations and live in a household with no handwashing facility. The rectangle depicts the percentage of children who received at least one dose of BCG, DPT, polio, or MCV and live in a household with handwashing facility.
